# Actovegin® reduces PMA-induced inflammation on human cells

**DOI:** 10.1007/s00421-020-04398-2

**Published:** 2020-05-23

**Authors:** Franz-Xaver Reichl, Christof Högg, Fangfang Liu, Markus Schwarz, Daniel Teupser, Reinhard Hickel, Wilhelm Bloch, Helmut Schweikl, Peter Thomas, Burkhard Summer

**Affiliations:** 1 Department of Conservative Dentistry and Periodontology, University Hospital, LMU Munich, Goethestr. 70, 80336 Munich, Germany; 2 Institute for Laboratory Medicine, University Hospital, LMU Munich, Munich, Germany; 3 Molecular and Cellular Sport Medicine, German Sport University, Cologne, Germany; 4 Department of Conservative Dentistry and Periodontology, University Hospital, Regensburg, Germany; 5 Department of Dermatology and Allergy, University Hospital, LMU Munich, Munich, Germany

**Keywords:** Sports, PMA, LPS, ROS, IL1-beta, Human PBMCs

## Abstract

**Purpose:**

The effect of Actovegin® was investigated on PMA- and LPS-induced human peripheral blood mononuclear cells (PBMCs).

**Methods:**

PBMCs (1 × 10^6^ cells/ml) from five blood donors (2 f, 3 m; 45–55 years) were grown in medium and exposed to Actovegin® in the presence or absence of PMA or LPS. Supernatants were collected to assess the concentration of cytokines (TNF-α, IL-1beta, IL-6 and IL-10). The reactive oxygen species (ROS) were assessed by a ROS-Glo^TM^ H_2_O_2_ assay.

**Results:**

Stimulation of cells by PMA or LPS (without Actovegin®) significantly increased the secretion of IL-1beta, IL-6, IL-10 and TNF-α from PBMCs, compared to controls. Pre-treatment of cells with Actovegin® (1, 5, 25, 125 µg/ml) plus PMA significantly decreased the secretion of IL-1beta from PBMCs, compared to controls (PMA without Actovegin®). In contrast, addition of Actovegin® (1, 5, 25, 125 and 250 µg/ml) plus LPS did not alter the IL-1beta production, compared to controls (LPS without Actovegin®). TNF-α, IL-6 and IL-10 do not contribute to the reduction of inflammatory reactions with Actovegin®.

**Conclusions:**

Actovegin® can reduce the PMA-induced IL-1beta release and the ROS production from PBMCs. These findings may help to explain the clinically known positive effects of Actovegin® on athletic injuries with inflammatory responses (e.g., muscle injuries, tendinopathies).

## Introduction

Actovegin® is a medical drug obtained from natural calf blood. Over 60 years, many medical indications are treated by Actovegin®, e.g., acute stroke (Boiarinov et al. 1998; Derev’yannykh et al. 2008) or postpartum hemorrhage (intravenous infusion) (Appiah 2002), skin ulcers (topical medication) (Biland et al. 1985), and long bone fractures (intra-arterial infusion) (Buchmayer et al. 2011), malfunction of the blood circulation in the brain and trophic disturbances (e.g., ischemic insult, craniocerebral injury) (Somogyi et al. 1979), impairment of peripheral blood circulation (e.g., angiopathy and ulcus cruris) (Buchmayer et al. 2011; Chanh et al. 1980; Lanner and Argyropoulos 1975), wound healing issues (e.g., torpid wounds, decubitus) (Buchmayer et al. 2011; Mochida et al. 1989; Neinhardt 1967; Schonwald et al. 1991) and mucosal lesions after radiation (Basu et al. 1985; Bauer and Locker 1974; Beetz et al. 1996; Buchmayer et al. 2011; Spessotto et al. 1993).

Muscle injury incidence varies from 30 to 55%; therefore, it is one of the most common sports-related injuries (Ekstrand et al. 2011; Jarvinen et al. 2000; Verrall et al. 2001). Twelve percent of all muscle injuries are hamstring injuries, which are 2.5 times more frequent than, for example, quadriceps injuries (Askling et al. 2003; Woods et al. 2004). It has been shown that muscle healing can be promoted by administration of anti-inflammatory drugs (Abramson and Weissmann 1989). However, anti-inflammatory drugs also can have an adverse effect on the entire healing process (Obremsky et al. 1994; Shen et al. 2005). Moreover, a recent systematic review illustrates the potential myotoxicity of local anesthetics and non-steroidal anti-inflammatory drug injection, while there is no evidence that Actovegin® has such a side effect (Reurink et al. 2014). A variety of treatments such as growth factor injection therapy is still very experimental and has shown initial results in some pilot studies; however, due to their performance enhancing and anabolic properties, they are prohibited by the World Anti-Doping Agency (WADA) (2019).

1990 Pfister and Koller first described intramuscular injection of Actovegin® as treatment of muscle injuries in a partially blinded case control study with 102 patients (Pfister and Koller 1990). Their study showed a reduction in recovery time in a treatment group of 5.5 weeks, compared to 8.3 weeks for the control group (Pfister and Koller 1990). However, in this study, the diagnosis of specific muscle injuries was only based on clinical findings and was not graded according to imaging, e.g., Magnetic Resonance Imaging (MRI). Furthermore, Actovegin® was mixed with anesthetics before injection resulting in pharmacodynamic and pharmacokinetic alterations (Pfister and Koller 1990).

In vivo and in vitro studies suggest that Actovegin® contains some active components, although they were not identified (Biland et al. 1985; Pforringer et al. 1994; Wright-Carpenter et al. 2004; Yaffe and Saxel 1977). In a previous in vitro study, an enhancement of the mitochondrial oxidative phosphorylation was registered in permeabilized human muscle fibers (obtained from overweight and untrained subjects) acutely exposed to Actovegin® (Sondergard et al. 2016). Hitherto, the effect of stand-alone Actovegin® therapy in muscle precursor cells highly relevant in skeletal muscle regeneration was not investigated in vivo and/or in vitro studies. To investigate effects of various substances/solutions on muscle precursor cell proliferation, optimal experimental condition are represented by C2C12 muscle cells (Yaffe and Saxel 1977). In our recent study, for the first time the effect of a stand-alone Actovegin® addition on the proliferation of C2C12 muscle cells was described, and Actovegin® increased the proliferation of muscle cells (Reichl et al. 2017). Furthermore, in this study the ingredients of Actovegin® were identified and the active substances on muscle proliferation were discussed in detail (Reichl et al. 2017).

There is much media attention and there are many anecdotal beliefs regarding Actovegin® injection therapy. In the lay press, controversial discussions between proponents and opponents have been published in recent years regarding the use of Actovegin® in high performance athletes. In our recent study a risk assessment was given and it could be demonstrated that Actovegin® may not be classified as a doping agent (Reichl et al. 2017). Furthermore, some clinical studies for Actovegin® confirm its safety (Maillo 2008; Pforringer et al. 1994; Ziegler et al. 2009).

The effect of anti-inflammatory drugs on muscle regeneration is controversial discussed. It was described that anti-inflammatory drugs can improve muscle regeneration by reducing degeneration and inflammation (Abramson and Weissmann 1989); however, in other studies, it was described that anti-inflammatory drugs are not conducive to the healing process (Obremsky et al. 1994; Shen et al. 2005).

Human mononuclear cells of the peripheral blood (PBMCs) are a useful tool to investigate anti-inflammatory effects of substances or antigens, as these immune cells of the peripheral blood actively participate in the healing processes after inflammation (Summer et al. 2010; Thomas et al. 2013).

In the present study the effect of Actovegin® was investigated on inflammation reactions on human PBMCs.

Our hypothesis was that Actovegin® has an anti-inflammatory effect on human cells.

## Materials and methods

### Cell culture and cell exposure

Stimulation assays were performed according to Summer et al. (2010), with the optimizations reported by Ständer et al. (2017). Heparinized blood was taken from anonymized healthy blood donors (2 females, 3 males, 45 − 55 years, non-smokers, no drug administration, no medication). After isolation of peripheral blood mononuclear cells (PBMCs) by density centrifugation, PBMCs of each blood donor were separately cultivated either with Phorbol 12-myristate 13-acetate (PMA) (1 µg/ml, Sigma-Aldrich, Munich, Germany) or with lipopolysaccharide (LPS, 10 ng/ml) with or without Actovegin® in different concentrations in quadruplicate. Cells (1 × 10^6^ cells/ml) were grown in RPMI 1640 medium in 96-well plates at 37 °C for 24 h. Actovegin® (200 mg/5 ml; Lot-No. 10946788; Takeda Austria GmbH, Linz, Austria) was directly diluted in cell culture medium to 0–1–5–25–125–250 µg/ml exactly as described in a recent investigation on muscle cell proliferation (Reichl et al. 2017). Cell cultures were exposed to these Actovegin® concentrations in the presence or absence of PMA (1 µg/ml) for 24 h. After the exposure, culture supernatants were collected for cytokine analysis.

The five blood donors were healthy individuals with normal blood cell counts with 1500–3000 lymphocytes/µl blood and 280–500 monocytes/µl blood. As for the healing process after inflammation all blood cells support the healing process we wanted to simulate a quite physiological situation with all mononuclear blood cells as already described in our previous study (Summer et al. 2018).

### Cytokine assays

The amount of IL-1beta, IL-6, IL10 and TNF-α was assessed by a multiplex cytometric bead assay according to the manufacturer´s protocol (BD, Biosciences, Heidelberg, Germany) in a FACS Canto flow cytometer.

### ROS assessment

The reactive oxygen species (ROS) were assessed in an identical experimental assay by ROS-Glo™ H_2_O_2_ Assay (Promega, Mannheim, Germany) according to the manufacturer protocol.

### Statistical analyses

Individual data from independent experiments were now summarized as medians (25–75% quartiles). Statistically significant differences between mean values were calculated using now the one-way ANOVA-Test followed by Games Howell post hoc test (SPSS Statistics 23, IBM, Armonk, NY, USA). The level of statistical significance was set to *p *< 0.05.

## Results

Stimulation with PMA (1 µg/ml) (without Actovegin®) significantly (*p* < 0.05) increased the secretion of IL-1beta, IL-6, IL-10 and TNF-α from PBMCs, compared to controls without PMA (Table 1).

**Table 1:** Cytokine production by human peripheral blood mononuclear cells (PBMCs) after stimulation with Phorbol 12-myristate 13-acetate (PMA) or lipopolysaccharide (LPS) for 24 h (without Actovegin®) (mean ± sem, *n *= 5)

	IL-1beta	IL-6	IL-10	TNFa
Medium	0.0 pg/ml	0.0 pg/ml	0.0 pg/ml	0.0 pg/ml
PMA	2920.98 pg/ml ± 1010.66	2320.45 pg/ml ± 1753.71	2.98 pg/ml ± 1.02	3005.15 pg/ml ± 596.21
LPS	3623.13 pg/ml ± 381.2	11316.4 pg/ml ± 3367.12	314.5 pg/ml ± 70.8	2520.25 pg/ml ± 937.1

Similarly, stimulation with LPS (10 µg/ml) (without Actovegin®) significantly (*p* < 0.05) increased the secretion of IL-1beta, IL-6, IL-10 and TNF-α from PBMCs, compared to controls without LPS (Table 1).

**Fig. 1:**
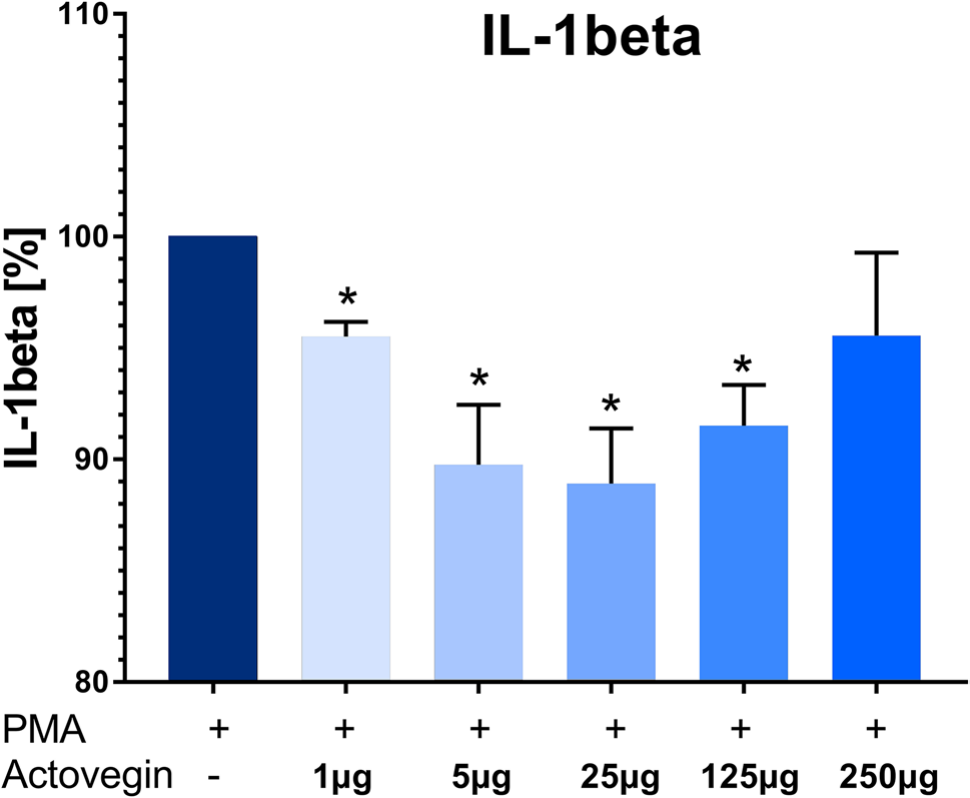
IL-1beta production by human peripheral blood mononuclear cells (PBMCs) after stimulation with Phorbol 12-myristate 13-acetate (PMA) (set as 100%) and addition of Actovegin® in 5 different concentrations (mean ± sem, *n *= 5; *p < 0.05)

Addition of Actovegin® (1, 5, 25, 125 µg/ml) plus PMA (1 µg/ml) significantly (*p* < 0.05) decreased the secretion of IL-1beta from PBMCs, compared to the control condition with PMA only (without Actovegin®) (Fig. 1).

**Fig. 2:**
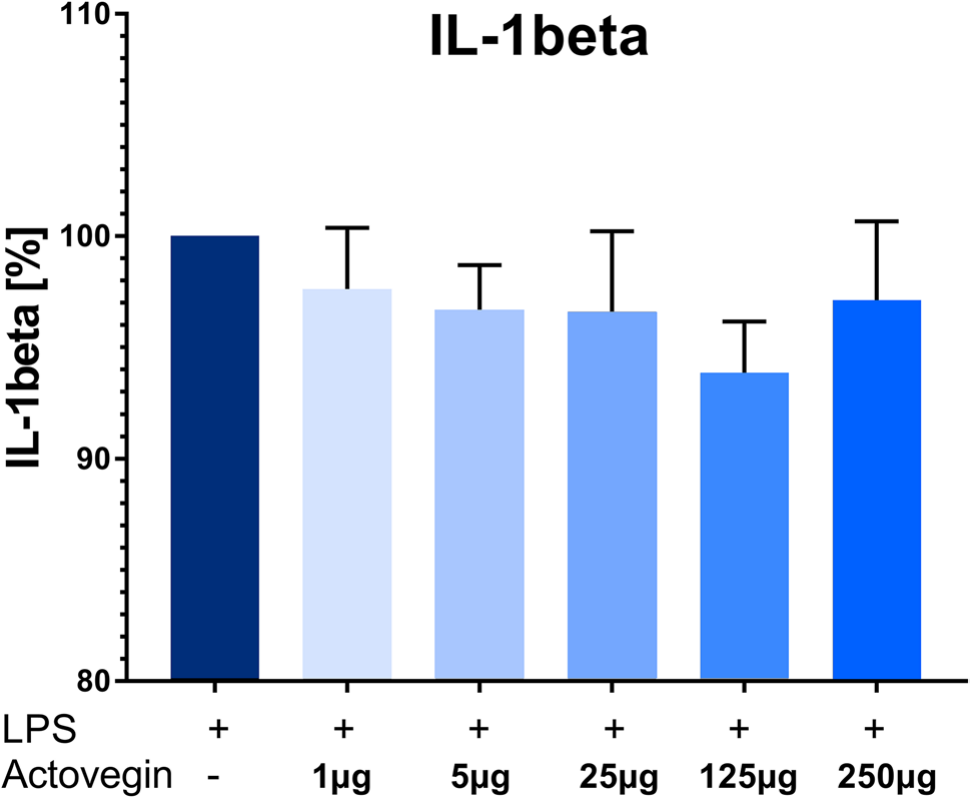
IL-1beta production by human peripheral blood mononuclear cells (PBMCs) after stimulation with lipopolysaccharide (LPS) (set as 100%) and addition of Actovegin® in five different concentrations (mean ± sem, *n *= 5)

In contrast, addition of Actovegin® (1, 5, 25, 125 and 250 µg/ml) plus LPS (10 ng/ml) did not result in a change of IL-1beta production, compared to the control condition with LPS only (without Actovegin®) (Fig. 2).

IL-6, IL-10 and TNFα production were not significantly changed by the addition of Actovegin® (1, 5, 25, 125 and 250 µg/ml) after stimulation with PMA or LPS (data not shown).

**Fig. 3:**
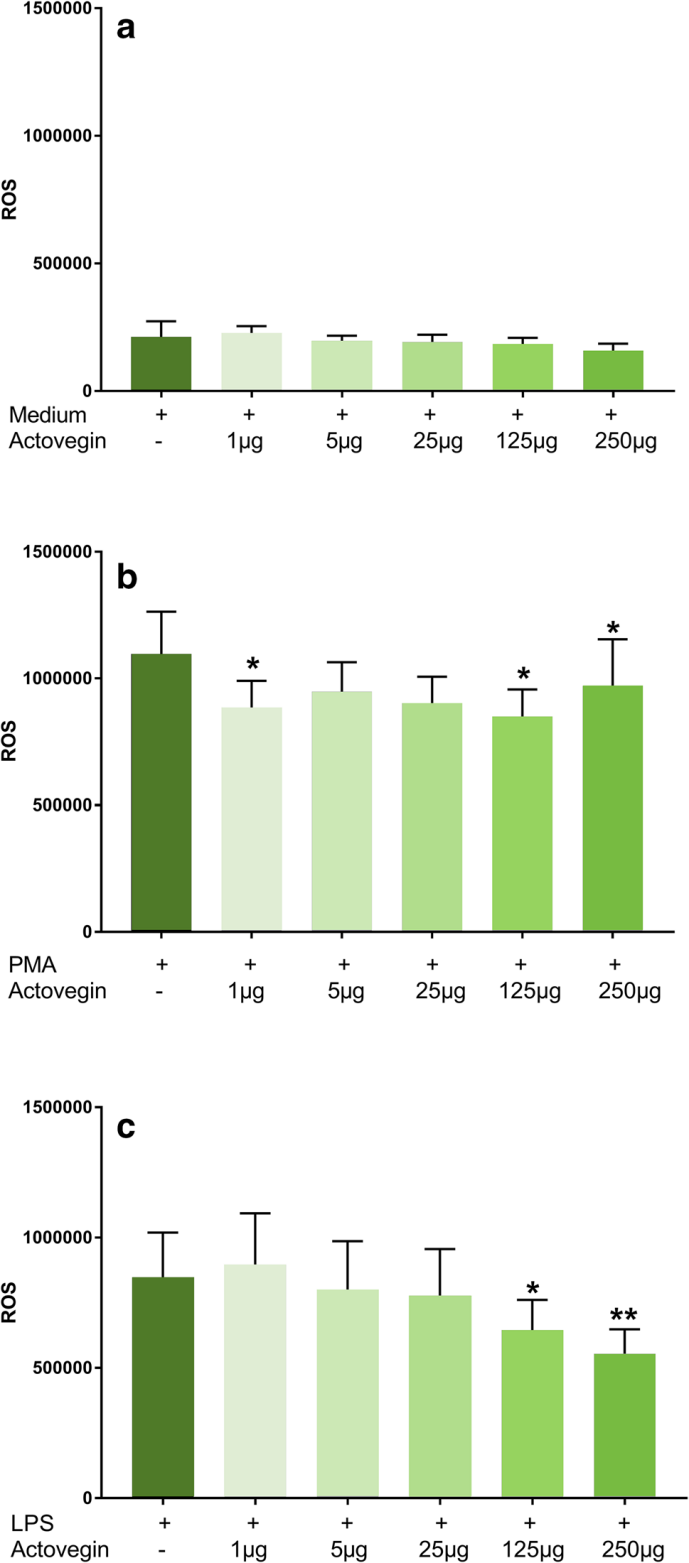
ROS production by human peripheral blood mononuclear cells (PBMCs) after stimulation with Actovegin® alone (Fig. 3a), Phorbol 12-myristate 13-acetate (PMA) and Actovegin® (Fig. 3b) and lipopolysaccharide (LPS) and Actovegin® (Fig. 3c) (mean ± sem, *n* = 5; *p < 0.05, **p < 0.01)

Addition of Actovegin® without PMA or LPS stimulation had no effect on ROS production, compared to controls (without Actovegin® and with medium only) (Fig. 3a).

Addition of Actovegin® (1, 125 and 250 µg/ml) significantly reduced the PMA-induced ROS production, compared to the control condition with PMA only (without Actovegin®) (Fig. 3b).

Addition of Actovegin® (125 and 250 µg/ml) significantly reduced the LPS-induced ROS production, compared to control condition with LPS only (without Actovegin®) (Fig. 3c).

## Discussion

Until today, the effect of anti-inflammatory drugs on muscle healing after injury is controversially discussed: Anti-inflammatory drugs can improve muscle regeneration by reducing degeneration and inflammation (Abramson and Weissmann 1989), in contrast, it is described that anti-inflammatory drugs are not conducive to the healing process (Obremsky et al. 1994; Shen et al. 2005).

PBMCs are a useful tool to investigate inflammation reactions. PBMCs (i.e., lymphocytes and monocytes) play a central role in muscle repair and regeneration during the inflammation that follows muscle injury. Therefore, we used in our model the PBMCs as described in previous study (Tidball 1995).

Here, we study the effect of Actovegin® in Phorbol 12-myristate 13-acetate (PMA)- and lipopolysaccharide (LPS)-induced inflammation on human PBMCs. LPS acts as the prototypical endotoxin, because it binds receptor complexes in many cell types, which promotes the secretion of pro-inflammatory cytokines (Abbas and Lichtman 2007). In our study the addition of Actovegin® did not influence the LPS-induced release of interleukines.

PMA is also a potent tumor promoter via activation of the signal transduction enzyme protein kinase C (PKC) (Castagna et al. 1982; Blumberg 1988; Niedel et al. 1983). The PKC pathway is critically involved in function and development of B cells, as well (Ghamlouch et al. 2014). PMA is routinely used as an inducer for endogenous superoxide production, as well, since it has been demonstrated to induce superoxide as the major ROS (Huang et al. 2014; Swindle et al. 2002). ROS are generated by NADPH oxidase in a process called the respiratory burst. This key enzyme catalyzes the generation of superoxide and hydrogen peroxide using electron provided by the hexose monophosphate shunt (Bastian and Hibbs 1994; Segal 1995; Jones et al. 2000). NADPH oxidase of phagocytic cells can also be activated by protein kinase C agonists such as PMA (Segal and Abo 1993).

PMA initiates acute inflammatory responses in mammals that are characteristic for the host reaction to tissue injury and/or infection (Basyreva et al. 2016).

Tissue response following injury or surgical trauma involves participation of blood-derived components, cytokines and growth factors. PMA induces high cytokine responses in PBMCs after 24 h stimulation including pro-inflammatory cytokines as IL-1beta, IL-6 and TNF-α but also anti-inflammatory cytokines as for example IL-10 and is, therefore, widely used as a suitable positive control in in vitro PBMCs stimulation experiments, where high cytokine production is expected and where freshly isolated PBMCs are used (David et al. 2003; Jeurink et al. 2008).

In the present study the effect of Actovegin® on the release of the important inflammatory cytokines IL-1 beta, IL-6, IL-10 and TNF-α (Wu et al. 2003) was investigated in PMA- and LPS-stimulated human PBMCs. The PMA-induced increase in the secretion of the pro-inflammatory cytokine IL-1 beta was dose dependently inhibited by addition of Actovegin®.

Oxidative damage plays a key role in inflammatory reactions and can induce several injuries (e.g., septic shock, atherosclerose) induced by PMA and/or LPS which are known to enhance the formation of reactive oxygen species (ROS) (Glebov and Zinchuk 2006; Swindle et al. 2002). PMA/LPS can induce the activation of TLR4 in the cell wall, which can induce ROS, e.g., superoxide (Swindle et al. 2002).

In the present study a dose dependent decrease of IL-1beta was observed after addition of Actovegin® only with PMA. Therefore, it could be shown that Actovegin® has an anti-inflammatory effect. Additionally, it could be shown that the addition of Actovegin® decreased the ROS production.

Two questions are arising from our results: (1) What is the mechanism of Actovegin’s^®^ effect of ROS formation? (2) Why is there a significant effect of Actovegin® on IL-1beta production in PMA-stimulated, but not in LPS-stimulated cells, i.e., what is the difference between LPS and PMA stimulation of PBMCs?

### Ad 1 (ROS formation)

Possible explanations are, that Actovegin® may support the antioxidative systems in the cells consisting of enzymatic and non-enzymatic antioxidants. Enzymes like catalase and substances such as glutathione as the major non-enzymatic antioxidant reduce oxidative stress by decreasing the levels of reactive oxygen species (ROS). Concentrations of ROS are crucial in the formation of inflammatory cytokines such as IL-1beta and IL-6 as well (Lavieri et al. 2016). In our recent study high levels of cystathionine in Actovegin® were detected, compared to the human adult serum/plasma (Reichl et al. 2017). Cystathionine is a precursor of cysteine synthesis which in turn is a component of the tripeptide glutathione. Both cysteine and glutathione contain sulfhydryl-groups and can, therefore, effectively act as antioxidants (Paul et al. 2018). Thus, an antioxidative effect of Actovegin® may be at least in part explained by the high availability of cystathionine.

Another antioxidative system represents the enzyme catalase (oxidoreductase). Human catalase is a peroxisomal enzyme. It is implicated in inflammation, ethanol metabolism, apoptosis, aging and cancer (Goodsell 2004). It is a common enzyme found in nearly all living organisms predominantly in the liver, kidney and erythrocytes (Goodsell 2004). It is a very important enzyme in protecting the cell from oxidative damage by reactive oxygen species (ROS). Catalase has one of the highest turnover numbers, each second one molecule can convert millions of hydrogen peroxide molecules to water and oxygen (Goodsell 2004). Superoxide is also biologically toxic and is employed by the immune system to kill invading microorganisms. Superoxide can be converted in cells into hydrogen peroxide which can further be catalyzed by catalase. Catalase is a tetramer of four polypeptide chains, each over 500 amino acids long, with four iron-containing heme groups that allow the enzyme to react with the ROS. Most intersubunit contacts are confined to the amino-terminal arms and the wrapping domains. The amino-terminal domain becomes almost completely buried between neighboring subunits in the tetramer. There are numerous salt bridges at the interfaces between monomers, mostly involving glutamic acid, asparagine acid, and arginine (Boon et al.). In our recent study high levels of glutamic acid, asparagine acid, and arginine in Actovegin® were detected, compared to the human adult serum/plasma (Reichl et al. 2017). The antioxidative effect of Actovegin® may be explained by the high availability of these Actovegin® ingredients. Therefore, the observed antioxidative effect of Actovegin® may be better understandable.

#### Ad 2 (differential effect after LPS vs. PMA stimulation)

LPS is a characteristic cell membrane component of Gram-negative bacteria, recognized by different immune cells—mainly monocytic cells—via Toll-like receptor 4 (TLR4) (Wassenaar and Zimmermann 2018). After LPS binding to TLR4, intracellular signaling pathways result in secretion of IL-1β after utilizing the NOD-like receptor containing-pyrin domain 3 (NLRP3) inflammasome and the subsequent cleavage of pro-caspase-1 into caspase-1 (Wynick et al. 2016).

However, this effect seems not be discriminative between LPS and PMA, since PMA has been shown to exert its well-known effect on monocyte differentiation via caspase‐1 activation (Niu et al. 2017). Moreover, there is another mechanistic similarity between LPS and PMA on monocytic cells: Ribeiro and colleagues could demonstrate that activated protein kinase C (PKC) further activates mTORC1/S6K pathway in a similar effect observed to LPS (Ribeiro et al. 2018). This observation is important for the interpretation of our results, since PMA is a potent activator of PKC (Leonard et al. 2015).

Altogether, it appears that the effect of LPS and PMA on monocytic cells seems to be similar. Therefore, the distinct effect of PMA on IL-1beta secretion in our PBMC model may not be attributable to the effect on monocytes. In contrast, there is quite distinct effect of PMA compared to LPS on lymphocytes: PMA is a well-established inducer of the differentiation of CLL B cells into plasmacytoid cells (Ghamlouch et al. 2014). In lymphocytes derived from healthy individuals, PMA stimulation results in a strong increase of CD23 phosphorylations, equal to the CD23 phosphorylation observed in B cells of patients with an active form of CLL (Madarova et al. 2018). To the best of our knowledge, there is no similar effect of LPS on lymphocytes.

The selective effect of Actovegin® on IL-1beta secretion in PMA-stimulated, but not in LPS-stimulated PBMCs may, therefore, be due to its effect on lymphocytes (most probably B cells) and not on monocytes.

PMA is known to promote tumor outgrowth through activation of serine/threonine-specific protein kinases. These serine/threonine-specific protein kinases appear to be responsible for the abnormal phosphorylations of CD23 protein in healthy B cells, as well (Madarova et al. 2018). Antagonism of this mechanism might be relevant for the effect of Actovegin® as well.

In this study a significant decrease was found only for the pro-inflammatory cytokine IL1-beta after addition of Actovegin® with PMA, compared to the release without Actovegin®, but not for the release of the pro-inflammatory cytokines IL-6 and TNF-α and for the anti-inflammatory IL-10. Therefore, the next question arising from our results concerns the difference between the investigated cytokines. IL1-beta is a key inflammatory mediator driving the host response to infection, injury, and disease. IL1-beta driven inflammation has often disastrous consequences, and thus represents a therapeutic target (Dinarello 2011). Caspase 1 is activated by recruitment to a molecular platform called an inflammasome (Schroder and Tschopp 2010) and caspase 1 is considered to belong to the inflammatory group (Siegel 2006).

Inhibition of caspase 1 would be anti-inflammatory by preserving cell viability and, therefore, limiting the release of pathogen-associated molecular pattern (PAMPs), consequently resulting in less inflammation (Denes et al. 2012). Inhibition or deletion of caspase 1 improves, e.g., outcome after myocardial infarction (Pomerantz et al. 2001; Frantz et al. 2003; Holly et al. 1999).

Studies describing clinical use of anti-IL-1 therapies focus almost on the use of biologicals such as IL-1Ra (anakinra) or anti-IL-1b antibodies such as canakinumab and other substances (Lopez-Castejon and Brough 2011) but were not successful (Dinarello 2011).

ICEberg is a protein that inhibits generation of IL-1beta by interacting with caspase-1 (Druilhe et al. 2001). ICEberg is induced in human cells by pro-inflammatory stimuli, suggesting that it may be part of a negative feedback loop. Consistent with this, enforced retroviral expression of ICEberg inhibits IL-1beta generation (Wu et al. 2003). The distribution of surface charge is complementary to the homologous prodomain of caspase-1, suggesting that charge–charge interactions mediate binding of ICEberg to the prodomain of caspase-1 (Humke et al. 2000).

Humke et al. (2000) detected in ICEberg in main domains (helix 1–6) the following aminoacids: Arginine (Arg), Lysine (Lys), Glutamic acid (Glu) and Asparagine acid (Asp). The surface of ICEberg contains three highly charged patches (Humke et al. 2000).

In our recent study it could be demonstrated that Actovegin® contains many physiological substances in significantly higher concentrations, compared to human adult serum (Reichl et al. 2017). The ICEberg aminoacids Arg, Lys, Glu, and Asp were found with 2-, 4-, 14-, and 14-fold higher concentrations, compared to human adult serum (Reichl et al. 2017). For the intact ICEberg synthesis and ICEberg function, these aminoacids are necessary and must be also available in the cells.

The significantly decreased IL-1beta release, after Actovegin® application may be explained by the successful synthesis of the enzyme ICEberg which is only possible by availability of these relevant aminoacids. Then ICEberg may powerful inhibit caspase 1 and may, therefore, result in an anti-inflammatory effect.

For an intact antioxidative and/or anti-inflammatory system with many proteins not only aminoacids are necessary, for the anabolic and catabolic pathways just like energy (e.g., ATP) and important inorganic substances (e.g., potassium, chloride, sodium, phosphate) are also necessary. ATP may be formed from increased availability and uptake of glucose. In the recent Actovegin® analysis for glucose a fourfold higher level, and for potassium, chloride, sodium, and phosphate up to tenfold higher levels were detected, compared to the corresponding substance levels in the adult human physiological serum/plasma (Reichl et al. 2017). Therefore, the observed anti-inflammatory effect of Actovegin® may be explained by the high availability of these Actovegin® ingredients in cells.

It is to note that TNF-α and IL-6 are also pro-inflammatory cytokines; however, the addition of Actovegin® with PMA or LPS did not lead to a decrease of the release of TNF-α or IL-6; therefore, TNF-α and IL-6 do not contribute to the reduction of inflammatory reactions with Actovegin®.

IL-10 is an anti-inflammatory cytokine. The addition of Actovegin® with PMA or LPS did not lead to an increase of the release of IL-10; therefore, IL-10 does also not contribute to the reduction of inflammatory reactions with Actovegin®.

It is mentioned that the transferability of in vitro results to the human physiological situation is limited but it is to note that this study has also a new direct relation to inflammations in sports medicine. Actovegin® is not only used in the above mentioned scopes of application and in skeletal muscle, but also as anti-inflammatory medication in skeletal muscle and in tendinopathies, e.g., on the patellar and achilles tendon (Reichl et al. 2017; Boiarinov et al. 1998; Derev’yannykh et al. 2008; Appiah 2002; Biland et al. 1985; Buchmayer et al. 2011; Somogyi et al. 1979; Chanh et al. 1980; Lanner and Argyropoulos 1975; Mochida et al. 1989; Neinhardt 1967; Schonwald et al. 1991; Basu et al. 1985; Bauer and Locker 1974; Beetz et al. 1996; Spessotto et al. 1993). Actovegin® is used as peritendinous injection (not intra-tendinous). Clinical experience indicate that inflammatory response and adhesions in the peritendinous tissue can be reduced with several injections of Actovegin® (Hotfiel et al. 2018). The present study was conducted to analyse if Actovegin® has an anti-inflammatory effect at all. The data support the clinical therapeutic findings and can help to explain how Actovegin® may work as a therapeutic agent when it is injected into inflamed tissue, e.g., around the patellar tendon, the achilles tendon or other locations that are mechanically inflamed.

## Conclusion

Our hypothesis is confirmed. Actovegin® exerts an anti-inflammatory effect, by dose- dependently diminishing the PMA-induced release of the pro-inflammatory interleukin Il-1beta in human PBMCs. This effect may be due to a specific effect on B cells. Moreover, we could demonstrate an anti-inflammatory effect by reduction of LPS- and PMA-induced ROS species. These results further indicate that Actovegin® can supply valuable components for the formation and function of an efficient antioxidative and/or anti-inflammatory system in cells, which may contribute to the reduction of an inflammation. These findings may also help to understand the positive effects of Actovegin® on inflammation injuries (right up to high performance athletes) and how Actovegin® may work as a therapeutic agent when it is injected into inflamed tissue.

## Data Availability

All data generated or analysed during this study are included in this published article.

## Abbreviations

LPS: Lipopolysaccharide
MRI: Magnetic Resonance Imaging
NADPH: Nicotinamide adenine dinucleotide phosphate
NLRP3: Nucleotide-binding oligomerization domain-like receptor containing-pyrin domain 3
PAMP: Pathogen-associated molecular pattern
PBMCs: Human peripheral blood mononuclear cells
PKC: Protein kinase C
PMA: Phorbol 12-myristate 13-acetate
ROS: Reactive oxygen species
TLR4: Toll-like receptor 4
WADA: World Anti-Doping Agency

## References

[CR1] Abbas AK, Lichtman AH (2007). Basic immunology: functions and disorders of the immune system.

[CR2] Abramson S, Weissmann G (1989). The mechanisms of action of nonsteroidal antiinflammatory drugs. Clin Exp Rheumatol.

[CR3] Appiah AK (2002). Treatment of severe primary postpartum hemorrhage with a deproteinized hemodialysate. Int J Gynaecol Obstet.

[CR4] Askling C, Karlsson J, Thorstensson A (2003). Hamstring injury occurrence in elite soccer players after preseason strength training with eccentric overload. Scand J Med Sci Sports.

[CR5] Bastian NR, Hibbs JB (1994). Assembly and regulation of NADPH oxidase and nitric oxide synthase. Curr Opin Immunol.

[CR6] Basu SK, Srinivasan MN, Chuttani K, Ghose A (1985). Evaluation of some radioprotectors by the survival study of rats exposed to lethal dose of whole body gamma radiation. J Radiat Res.

[CR7] Basyreva LY, Brodsky IB, Gusev AA, Zhapparova ON, Mikhalchik EV, Gusev SA, Shor DB, Dahan S, Blank M, Shoenfeld Y (2016). The effect of Intravenous Immunoglobulin (IVIG) on \textit{ex vivo} activation of human leukocytes. Hum Antibodies.

[CR8] Bauer D, Locker A (1974). The radioprotective effect of solcoseryl. Experientia.

[CR9] Beetz A, Machicao F, Ried C, Ruzicka T, Michel G (1996). Radioprotective effects of a protein-free hemodialysate in human epidermis. Skin Pharmacol.

[CR10] Biland L, Hurlimann F, Goor W, Korner WF, Kundig A, Madar G, Widmer LK, Ziegler WJ (1985). Treatment of venous ulcers. A multi-center randomized double-blind study. Vasa.

[CR11] Blumberg PM (1988). Protein kinase C as the receptor for the phorbol ester tumor promoters: sixth Rhoads memorial award lecture. Cancer Res.

[CR12] Boiarinov GA, Mukhina IV, Penknovich AA, Snopova LB, Zimin IuV, Balandina MV, Radaev AM, Skvortsova IE, Prodanets NN (1998). Mechanisms of actovegin effect on the central nervous system during postischemic period. Biull Eksp Biol Med.

[CR13] Buchmayer F, Pleiner J, Elmlinger MW, Lauer G, Nell G, Sitte HH (2011). Actovegin(R): a biological drug for more than 5 decades. Wien Med Wochenschr.

[CR14] Castagna M, Takai Y, Kaibuchi K, Sano K, Kikkawa U, Nishizuka Y (1982). Direct activation of calcium-activated, phospholipid-dependent protein kinase by tumor-promoting phorbol esters. J Biol Chem.

[CR15] Chanh PH, Chanh AP, Basile JP, Navarro C, Nguyen VT (1980). Cardiovascular activity of a deproteinized blood extract. Arzneimittelforschung.

[CR16] David KC, Brady MT, Weimer LK, Hellberg MR, Nixon JC, Graff G (2003). Characterization of the in vitro anti-inflammatory activity of AL-5898 and related benzopyranyl esters and amides. Inflammation.

[CR17] Denes A, Lopez-Castejon G, Brough D (2012). Caspase-1: is IL-1 just the tip of the ICEberg?. Cell Death Dis.

[CR18] Derev’yannykh EA, Bel’skaya GN, Knoll EA, Krylova LG, Popov DV (2008). Experience in the use of Actovegin in the treatment of patients with cognitive disorders in the acute period of stroke. Neurosci Behav Physiol.

[CR19] Dinarello CA (2011). Interleukin-1 in the pathogenesis and treatment of inflammatory diseases. Blood.

[CR20] Druilhe A, Srinivasula SM, Razmara M, Ahmad M, Alnemri ES (2001). Regulation of IL-1beta generation by Pseudo-ICE and ICEBERG, two dominant negative caspase recruitment domain proteins. Cell Death Differ.

[CR21] Ekstrand J, Hagglund M, Walden M (2011). Epidemiology of muscle injuries in professional football (soccer). Am J Sports Med.

[CR22] Frantz S, Ducharme A, Sawyer D, Rohde LE, Kobzik L, Fukazawa R, Tracey D, Allen H, Lee RT, Kelly RA (2003). Targeted deletion of caspase-1 reduces early mortality and left ventricular dilatation following myocardial infarction. J Mol Cell Cardiol.

[CR23] Ghamlouch H, Ouled-Haddou H, Guyart A, Regnier A, Trudel S, Claisse JF, Fuentes V, Royer B, Marolleau JP, Gubler B (2014). Phorbol myristate acetate, but not CD40L, induces the differentiation of CLL B cells into Ab-secreting cells. Immunol Cell Biol.

[CR24] Glebov AN, Zinchuk VV (2006). Prooxidant-antioxidant state of the organism during oxidative stress and correction of the l-arginine-NO system. Bull Exp Biol Med.

[CR25] Goodsell DS (2004). Catalase. Molecule of the Month.

[CR26] Holly TA, Drincic A, Byun Y, Nakamura S, Harris K, Klocke FJ, Cryns VL (1999). Caspase inhibition reduces myocyte cell death induced by myocardial ischemia and reperfusion in vivo. J Mol Cell Cardiol.

[CR27] Hotfiel T, Seil R, Bily W, Bloch W, Gokeler A, Krifter RM, Mayer F, Ueblacker P, Weisskopf L, Engelhardt M (2018). Nonoperative treatment of muscle injuries - recommendations from the GOTS expert meeting. J Exp Orthop.

[CR28] Huang R, Zhao L, Chen H, Yin RH, Li CY, Zhan YQ, Zhang JH, Ge CH, Yu M, Yang XM (2014). Megakaryocytic differentiation of K562 cells induced by PMA reduced the activity of respiratory chain complex IV. PLoS ONE.

[CR29] Humke EW, Shriver SK, Starovasnik MA, Fairbrother WJ, Dixit VM (2000). ICEBERG: a novel inhibitor of interleukin-1beta generation. Cell.

[CR30] Jarvinen TA, Kaariainen M, Jarvinen M, Kalimo H (2000). Muscle strain injuries. Curr Opin Rheumatol.

[CR31] Jeurink PV, Vissers YM, Rappard B, Savelkoul HF (2008). T cell responses in fresh and cryopreserved peripheral blood mononuclear cells: kinetics of cell viability, cellular subsets, proliferation, and cytokine production. Cryobiology.

[CR32] Jones RD, Hancock JT, Morice AH (2000). NADPH oxidase: a universal oxygen sensor?. Free Radic Biol Med.

[CR33] Lanner G, Argyropoulos (1975). Pharmacological effect of Solcoseryl on the metabolism of the brain Animal experiments and clinical research. Wien Med Wochenschr.

[CR34] Lavieri R, Rubartelli A, Carta S (2016). Redox stress unbalances the inflammatory cytokine network: role in autoinflammatory patients and healthy subjects. J Leukoc Biol.

[CR35] Leonard B, McCann JL, Starrett GJ, Kosyakovsky L, Luengas EM, Molan AM, Burns MB, McDougle RM, Parker PJ, Brown WL, Harris RS (2015). The PKC/NF-kappaB signaling pathway induces APOBEC3B expression in multiple human cancers. Cancer Res.

[CR36] Lopez-Castejon G, Brough D (2011). Understanding the mechanism of IL-1beta secretion. Cytokine Growth Factor Rev.

[CR37] Madarova M, Mucha R, Hresko S, Makarova Z, Gdovinova Z, Szilasiova J, Vitkova M, Guman T, Stecova N, Dobransky T (2018). Identification of new phosphorylation sites of CD23 in B-cells of patients with chronic lymphocytic leukemia. Leuk Res.

[CR38] Maillo L (2008). Anaphylactic shock with multiorgan failure in a cyclist after intravenous administration of Actovegin. Ann Intern Med.

[CR39] Boon EM, Downs A, Marcey D Catalase: H2O2: H2O2 Oxidoreductase. http://biology.kenyon.edu/BMB/Chime/catalase/frames/cattx.htm. Accessed Apr 2018

[CR40] Mochida H, Kikuchi T, Tanaka H, Ikeda A, Fujii Y, Sasamura T (1989). Influence of Actovegin® containing infusion solutions on wound healing and function of the intestinal tract in rats. Pharmacol Ther.

[CR41] Neinhardt J (1967) Extra- und intraorale Wundheilung. Dissertation/PhD Thesis, Universitäts- und Poliklinik für Zahn-, Mund-, und Kieferkrankheiten Würzburg,

[CR42] Niedel JE, Kuhn LJ, Vandenbark GR (1983). Phorbol diester receptor copurifies with protein kinase C. Proc Natl Acad Sci USA.

[CR43] Niu Z, Tang J, Zhang W, Chen Y, Huang Y, Chen B, Li J, Shen P (2017). Caspase-1 promotes monocyte-macrophage differentiation by repressing PPARgamma. FEBS J.

[CR44] Obremsky WT, Seaber AV, Ribbeck BM, Garrett WE (1994). Biomechanical and histologic assessment of a controlled muscle strain injury treated with piroxicam. Am J Sports Med.

[CR45] Paul BD, Sbodio JI, Snyder SH (2018). Cysteine metabolism in neuronal redox homeostasis. Trends Pharmacol Sci.

[CR46] Pfister A, Koller W (1990). Treatment of fresh muscle injury. Sportverletz Sportschaden.

[CR47] Pforringer W, Pfister A, Kuntz G (1994). The treatment of achilles paratendinitis: results of a double-blind, placebo-controlled study with a deproteinized hemodialysate. Clin J Sport Med.

[CR48] Pomerantz BJ, Reznikov LL, Harken AH, Dinarello CA (2001). Inhibition of caspase 1 reduces human myocardial ischemic dysfunction via inhibition of IL-18 and IL-1beta. Proc Natl Acad Sci USA.

[CR49] Reichl FX, Holdt LM, Teupser D, Schutze G, Metcalfe AJ, Hickel R, Hogg C, Bloch W (2017). Comprehensive Analytics of Actovegin(R) and Its Effect on Muscle Cells. Int J Sports Med.

[CR50] Reurink G, Goudswaard GJ, Moen MH, Weir A, Verhaar JA, Tol JL (2014). Myotoxicity of injections for acute muscle injuries: a systematic review. Sports Med.

[CR51] Ribeiro MC, Peruchetti DB, Silva LS, Silva-Filho JL, Souza MC, Henriques MDG, Caruso-Neves C, Pinheiro AAS (2018). LPS Induces mTORC1 and mTORC2 Activation During Monocyte Adhesion. Front Mol Biosci.

[CR52] Schonwald D, Sixt B, Machicao F, Marx E, Haedenkamp G, Bertsch S (1991). Enhanced proliferation of coronary endothelial cells in response to growth factors is synergized by hemodialysate compounds in vitro. Res Exp Med (Berl).

[CR53] Schroder K, Tschopp J (2010). The inflammasomes. Cell.

[CR54] Segal AW (1995). The NADPH oxidase of phagocytic cells is an electron pump that alkalinises the phagocytic vacuole. Protoplasma.

[CR55] Segal AW, Abo A (1993). The biochemical basis of the NADPH oxidase of phagocytes. Trends Biochem Sci.

[CR56] Shen W, Li Y, Tang Y, Cummins J, Huard J (2005). NS-398, a cyclooxygenase-2-specific inhibitor, delays skeletal muscle healing by decreasing regeneration and promoting fibrosis. Am J Pathol.

[CR57] Siegel RM (2006). Caspases at the crossroads of immune-cell life and death. Nat Rev Immunol.

[CR58] Somogyi E, Sotonyi P, Nemes A (1979). The effects of a deproteinized blood extract on the myocardial changes developing during experimentally induced intermittent hypoxia. Arzneimittelforschung.

[CR59] Sondergard SD, Dela F, Helge JW, Larsen S (2016). Actovegin, a non-prohibited drug increases oxidative capacity in human skeletal muscle. Eur J Sport Sci.

[CR60] Spessotto P, Dri P, Baschong W, Mittenzwei H, Patriarca P (1993). Effect of a protein-free dialysate from calf blood on human monocyte differentiation in vitro. Arzneimittelforschung.

[CR61] Stander S, Oppel E, Thomas P, Summer B (2017). Evaluation of lymphocyte transformation tests as compared with patch tests in nickel allergy diagnosis. Contact Dermatitis.

[CR62] Summer B, Paul C, Mazoochian F, Rau C, Thomsen M, Banke I, Gollwitzer H, Dietrich KA, Mayer-Wagner S, Ruzicka T, Thomas P (2010). Nickel (Ni) allergic patients with complications to Ni containing joint replacement show preferential IL-17 type reactivity to Ni. Contact Dermatitis.

[CR63] Summer B, Stander S, Thomas P (2018). Cytokine patterns in vitro, in particular IL-5/IL-8 ratio, to detect patients with nickel contact allergy. J Eur Acad Dermatol Venereol.

[CR64] Swindle EJ, Hunt JA, Coleman JW (2002). A comparison of reactive oxygen species generation by rat peritoneal macrophages and mast cells using the highly sensitive real-time chemiluminescent probe pholasin: inhibition of antigen-induced mast cell degranulation by macrophage-derived hydrogen peroxide. J Immunol.

[CR65] Thomas P, Iglhaut G, Wollenberg A, Cadosch D, Summer B (2013). Allergy or tolerance: reduced inflammatory cytokine response and concomitant IL-10 production of lymphocytes and monocytes in symptom-free titanium dental implant patients. Biomed Res Int.

[CR66] Tidball JG (1995). Inflammatory cell response to acute muscle injury. Med Sci Sports Exerc.

[CR67] Verrall GM, Slavotinek JP, Barnes PG, Fon GT, Spriggins AJ (2001). Clinical risk factors for hamstring muscle strain injury: a prospective study with correlation of injury by magnetic resonance imaging. Br J Sports Med.

[CR68] WADA (2019) Prohibited List January 2019. World Anti-Doping Agency

[CR69] Wassenaar TM, Zimmermann K (2018). Lipopolysaccharides in food, food supplements, and probiotics: should we be worried?. Eur J Microbiol Immunol (Bp).

[CR70] Woods C, Hawkins RD, Maltby S, Hulse M, Thomas A, Hodson A, Football Association Medical Research P (2004). The Football Association Medical Research Programme: an audit of injuries in professional football–analysis of hamstring injuries. Br J Sports Med.

[CR71] Wright-Carpenter T, Klein P, Schaferhoff P, Appell HJ, Mir LM, Wehling P (2004). Treatment of muscle injuries by local administration of autologous conditioned serum: a pilot study on sportsmen with muscle strains. Int J Sports Med.

[CR72] Wu X, Herndon DN, Wolf SE (2003). Growth hormone down-regulation of Interleukin-1beta and Interleukin-6 induced acute phase protein gene expression is associated with increased gene expression of suppressor of cytokine signal-3. Shock.

[CR73] Wynick C, Petes C, Tigert A, Gee K (2016). Lipopolysaccharide-Mediated Induction of Concurrent IL-1beta and IL-23 Expression in THP-1 Cells Exhibits Differential Requirements for Caspase-1 and Cathepsin B Activity. J Interferon Cytokine Res.

[CR74] Yaffe D, Saxel O (1977). Serial passaging and differentiation of myogenic cells isolated from dystrophic mouse muscle. Nature.

[CR75] Ziegler D, Movsesyan L, Mankovsky B, Gurieva I, Abylaiuly Z, Strokov I (2009). Treatment of symptomatic polyneuropathy with actovegin in type 2 diabetic patients. Diabetes Care.

