# Correction to: Actovegin® reduces PMA‑induced inflammation on human cells

**DOI:** 10.1007/s00421-021-04824-z

**Published:** 2021-10-15

**Authors:** Franz-Xaver Reichl, Christof Högg, Fangfang Liu, Markus Schwarz, Daniel Teupser, Reinhard Hickel, Wilhelm Bloch, Helmut Schweikl, Peter Thomas, Burkhard Summer

**Affiliations:** 1 Department of Conservative Dentistry and Periodontology, University Hospital, LMU Munich, Goethestr. 70, 80336 Munich, Germany; 2 Institute for Laboratory Medicine, University Hospital, LMU Munich, Munich, Germany; 3 Molecular and Cellular Sport Medicine, German Sport University, Cologne, Germany; 4 Department of Conservative Dentistry and Periodontology, University Hospital, Regensburg, Germany; 5 Department of Dermatology and Allergy, University Hospital, LMU Munich, Munich, Germany

## Correction to: European Journal of Applied Physiology (2020) 120:1671–1680 10.1007/s00421-020-04398-2

The article Actovegin® reduces PMA‑induced inflammation on human cells, written by Franz‑Xaver Reichl, Christof Högg, Fangfang Liu, Markus Schwarz, Daniel Teupser, Reinhard Hickel, Wilhelm Bloch, Helmut Schweikl, Peter Thomas and Burkhard Summer, was originally published Online First without Open Access. After publication in volume 120, issue 7, page 1671–1680 the author decided to opt for Open Choice and to make the article an Open Access publication. Therefore, the copyright of the article has been changed to © The Author(s) 2021 and this article is licensed under a Creative Commons Attribution 4.0 International License, which permits use, sharing, adaptation, distribution and reproduction in any medium or format, as long as you give appropriate credit to the original author(s) and the source, provide a link to the Creative Commons licence, and indicate if changes were made. The images or other third party material in this article are included in the article's Creative Commons licence, unless indicated otherwise in a credit line to the material. If material is not included in the article's Creative Commons licence and your intended use is not permitted by statutory regulation or exceeds the permitted use, you will need to obtain permission directly from the copyright holder. To view a copy of this licence, visit http://creativecommons.org/licenses/by/4.0/.

Open Access funding enabled and organized by Projekt DEAL.

The original article has been corrected.

